# How do hospital boards govern for quality improvement? A mixed methods study of 15 organisations in England

**DOI:** 10.1136/bmjqs-2016-006433

**Published:** 2017-07-08

**Authors:** Lorelei Jones, Linda Pomeroy, Glenn Robert, Susan Burnett, Janet E Anderson, Naomi J Fulop

**Affiliations:** 1 Department of Applied Health Research, University College London, London, UK; 2 The Florence Nightingale Faculty of Nursing& Midwifery, Kings College London, London; 3 Centre for Patient Safety and Service Quality, Imperial College London, London, UK

**Keywords:** Governance, Leadership, Quality Improvement

## Abstract

**Background:**

Health systems worldwide are increasingly holding boards of healthcare organisations accountable for the quality of care that they provide. Previous empirical research has found associations between certain board practices and higher quality patient care; however, little is known about how boards govern for quality improvement (QI).

**Methods:**

We conducted fieldwork over a 30-month period in 15 healthcare provider organisations in England as part of a wider evaluation of a board-level organisational development intervention. Our data comprised board member interviews (n=65), board meeting observations (60 hours) and documents (30 sets of board meeting papers, 15 board minutes and 15 Quality Accounts). We analysed the data using a framework developed from existing evidence of links between board practices and quality of care. We mapped the variation in how boards enacted governance of QI and constructed a measure of QI governance maturity. We then compared organisations to identify the characteristics of those with mature QI governance.

**Results:**

We found that boards with higher levels of maturity in relation to governing for QI had the following characteristics: explicitly prioritising QI; balancing short-term (external) priorities with long-term (internal) investment in QI; using data for QI, not just quality assurance; engaging staff and patients in QI; and encouraging a culture of continuous improvement. These characteristics appeared to be particularly enabled and facilitated by board-level clinical leaders.

**Conclusions:**

This study contributes to a deeper understanding of how boards govern for QI. The identified characteristics of organisations with mature QI governance seemed to be enabled by active clinical leadership. Future research should explore the biographies, identities and work practices of board-level clinical leaders and their role in organisation-wide QI.

## Introduction

There is growing international attention on the role of boards in supporting high-quality care.^1–3^ There are concerns that boards focus on finance and external performance standards at the expense of quality, and that board members lack the necessary experience, knowledge and skills for effective governance of quality.^3–5^ In the National Health Service (NHS) in England a number of high-profile public inquiries have questioned the effectiveness of board governance of quality.^6–8^ For example, the report of the Francis inquiry into serious failings of care at Mid Staffordshire NHS Foundation Trust found that the board did not listen to the concerns raised by patients and staff and ‘failed to tackle an insidious negative culture involving a tolerance of poor standards and disengagement from management and leadership responsibilities.’^8^ As a consequence, national regulatory bodies are currently seeking to strengthen board-level governance of quality.^9 10^ However, little is known about how boards enact governance of quality, what ‘good’ governance in relation to quality, and quality improvement (QI), looks like, and what organisational processes accomplish and sustain it.^3 11^ In this study, we used findings from previous empirical research to construct a framework to explore how governance of QI is enacted by boards. We use ‘quality improvement’ to refer to the use of systematic methods and tools to improve outcomes for patients on a continuous basis.^12^

### Background

Boards of NHS providers in England have tended to follow the unitary board model of the private sector. They typically have between 10 and 15 members comprising executive and non-executive functions. Overall leadership of the board resides with the chair. The Chief Executive leads the executive team with each member usually responsible for a specific function such as ‘finance’ or ‘human resources.’ Non-executives are charged with holding the management team to account. Executives and non-executives are expected to work collaboratively to develop strategy.^13^

NHS hospital boards operate within a highly politicised context with a ‘complex and unclear purpose and accountability structure.’^14^ Boards of NHS providers in England are ultimately accountable to the Secretary of State for Health. The majority of NHS provider organisations (142/242, 62%) are now ‘foundation trusts.’ Unlike non-foundation trusts, this type of organisation also incorporates horizontal lines of accountability, to members, and to the board of governors which appoint the non-executive directors. However, this form of local accountability is perceived as generally weak,^15^ and it has been argued that financial deficits and an increase in central planning guidance have narrowed the differences, in accountability processes, between foundation and non-foundation trusts.^16^

Previous research has found an association between certain board practices and the quality of care. A study of board chairs of US hospitals found that organisations which considered ‘quality of care’ to be one of the top two board priorities performed better on clinical quality. Other characteristics found to be associated with high-quality care were board chairs who were familiar with current performance, and board members with expertise in QI, and who had received formal training in QI.^17^ Other studies have found that higher quality care is associated with devoting a significant amount of time to quality issues at most board meetings, having a board quality committee, establishing strategic goals for QI, public dissemination of goals for QI, involvement in setting the quality agenda for the organisation, including a specific item on quality on board meeting agendas, and regularly reviewing quality performance to identify areas for improvement.^18–22^ Using a dashboard with national benchmarks for clinical quality, patient safety and patient satisfaction is associated with better quality care^18–20^; as is the approach of setting quality goals at a ‘theoretical ideal’ rather than average levels or national benchmarks.^21^

Since the report of the public inquiry into failings in care at Mid Staffordshire NHS Foundation Trust, the importance of involving staff and patients in formulating strategy is increasingly recognised in guidance to hospital boards.^23–25^ Guidance also highlights the importance of board dynamics, such as open and constructive debate.^13^

We used findings from this previous empirical research and guidance to construct a framework to explore how governance in relation to QI is enacted by boards. We then used our findings to develop a measure of organisational ‘maturity’ in relation to governance of QI. We applied this measure to the 15 organisations in our study to identify the processes adopted by boards with higher levels of maturity in their approach to governing for QI.

Our study differs from previous research on hospital boards that has employed board competency frameworks.^11 26^ Board competency frameworks relate to the overall performance of the board and, in the absence of empirical evidence on how boards fulfil their responsibilities, are largely normative.^3^ Moreover, their use to date has been based on self-assessment, which brings with it a risk of positive bias or limited insight.^27 28^ Our study makes a significant contribution to understanding how boards enact governance in relation to QI by using a framework based on research evidence and focused specifically on the governance of QI, and on external observation and assessment of real-world board practices.

## Methods

### Sample

We conducted fieldwork in 15 organisations (12 acute care providers, 2 mental health providers and 1 community care provider) as part of an evaluation of a board-level organisational development intervention (iQUASER).^29^ In England healthcare provider organisations may incorporate more than one hospital, but overseen by a single corporate board. For clarity we use the term ‘organisation’ to refer to the unit of analysis. Alongside the six organisations that received the intervention (‘participating organisations’), data were also collected from a comparator group (‘comparator organisations’) that included six organisations that matched each of those in the intervention group on the following dimensions: type of service provided (acute, community or mental health), foundation status, performance (as assessed by the English healthcare regulator) and number and location of sites. We also selected an additional three organisations (‘benchmarking organisations’) to reflect different performance levels (‘outstanding’, ‘good’ and ‘requires improvement’ as rated by a national regulator).

### Developing the framework

We used previous research and guidance to construct a framework to explore how hospital boards in England enact governance of QI. The framework approach was designed for studies with a defined research question of policy or practical relevance. It is well suited to multidisciplinary and mixed methods research and a homogenous data set.^30 31^ Our framework has eight dimensions, corresponding to the board characteristics identified in the literature as being associated with higher quality patient care (table 1).

**Table 1:** Framework for data collection and analysis

	Framework dimension	Evidence
1	QI as board priority	17–22
2	Using data for improvement	18–20
3	Familiarity with current performance	17 18
4	Degree of staff involvement	25
5	Degree of public/patient/carer involvement	25
6	Clear, systematic approach (clear and well-specified priorities, manageable number)	17 18 20
7	Balance between clinical effectiveness, patient experience and safety	18 20
8	Dynamics (how board members challenge/ask questions of each other)	13

QI, quality improvement.

### Data collection and analysis

Data were collected between March 2014 and November 2016. The public part of a board meeting was observed in all 15 organisations on two occasions, once in 2014 and once in 2015/2016 (60 hours observation). We also collected publicly available board meeting papers from the meetings we observed (30 sets) minutes from an additional board meeting for each organisation (15) and Quality Accounts (a mandatory report on the quality of services provided by an organisation and published annually). Interviews were undertaken with board members (up to six per organisation) in the six organisations that had agreed to participate in the evaluation and in one comparator and one benchmarking organisation which volunteered to take part in the interviews (interviews were not requested with the other organisations) (37 in 2014 and 28 in 2015/2016). We asked organisations to nominate five members, both executive and non-executive, including the non-executive director responsible for quality. Interviews were semistructured on the topic of governance of QI and covered the topics in our framework. Notes were handwritten at the time of board observations and then written up afterwards and stored electronically. Interviews were recorded and transcribed. All textual data, including board observations, interview transcripts and documents, were imported into NVIVO software for analysis.

Our analysis followed a number of stages. In the first stage we developed and tested a measure of an organisation’s ‘maturity’ in relation to governance of QI (QI maturity). This was an iterative process that involved successive rounds of analysis, team discussion, testing the emerging measure against the data and refinement.^32^ We used the entire data set to map the full range of board activities related to each dimension of our framework. For each activity a grading scheme (high/medium/low) was constructed to reflect observed variation in the data (table 2). This was an interpretative activity that drew on both the research literature and the three ‘benchmarking’ organisations.

**Table 2:** A measure of organisational maturity in relation to governing for QI (QI maturity)

Dimension	Rating
1. QI as a board priority	
Where does QI come in the agenda of board discussions?	High: Top of the agenda, for example, first item/included throughout/a specific standing item led by an executive director. Medium: In the middle of the meeting. Low: At the end of the meeting.
How much time is spent talking about QI?	High: Majority of board meeting related to QI. Medium: Some time given to QI. Low: Limited time at the board meeting related to QI.
Is time spent on QI elsewhere other than at the board meeting (eg, subcommittees)?	High: QI is dealt with predominantly at the subcommittee level with only points of escalation brought to the board meeting. Medium: QI is dealt with to some extent at the subcommittee level, for example, the subcommittee discussion appears to be fully reported to the board level. Low: QI is dealt with extensively at the board level, for example, discussion of all aspects of the subcommittee discussions.
Do board members undergo any formal QI training?	High: Formal training systematically undertaken. Medium: Training available and awareness of training but not necessarily taken up. Low: Limited or no QI training.
How much time is spent on QI relative to QA*?	High: Balance between QI and QA. QA monitored and when necessary actions taken that feed into QI. QI alongside QA, as an ongoing strand of discussions and focus. Medium: QA monitored and QI discussed but the balance is more towards assurance and there is not necessarily a well-managed link from QA to QI. Low: Predominantly QA.
2. Using data for improvement	
Does the Trust use QI-specific data?	High: QI data available and presented to board members. Medium: Some QI data available and/or some QI discussion relating to the quality data that is presented. Low: No QI data presented and no QI discussion relating to the quality data.
To what extent is the use of data proactive rather than reactive?	High: Regular and consistent use of data. Medium: Some evidence of using data and/or some awareness of the need to move to a more proactive approach. Low: Searching for data only when there is a perceived need.
Are data presented in a meaningful format?	High: Data are used, interpreted and discussed. Format is readable. Medium: Data in a useable format but minimal interpretation. Low: A lot of data presented but unreadable and limited interpretation or discussion.
Are data used to inform actions?	High: There are clear actions derived from data. Medium: Some evidence of actions being informed by data. Low: Limited evidence of actions being informed by data.
Are QI data linked to other data (eg, staffing levels, sickness absence, throughput)?	High: Data are clearly linked and discussions about QI take into account all the data available. Medium: Data are linked to some extent. Low: Each source of data considered in isolation.
Does the board consider a broad range of data (eg, quantitative alongside qualitative)?	High: Broad range of data considered, that is, both quantitative and qualitative data. Medium: Predominantly focused on quantitative data (or one type of data) but aware and/or working towards a broader use of data. Low: Focused solely or predominantly on quantitative data and no awareness of the need or usefulness of a broader range of data.
3. Familiarity with current performance	
Are the board looking at current performance frequently?	High: Monthly review of data, awareness and understanding of the data, for example, questions about the data are knowledgeable. Medium: Data reviewed less than once a month, lower awareness and understanding. Low: Data reviewed less often or not available. Limited understanding and awareness.
Does the board benchmark and compare with other organisations?	High: Comparative assessment with other organisations discussed frequently. Medium: Comparative assessments done but not frequent. Low: Limited comparative assessment with other organisations.
Is there an awareness of the data available and where data needed to be improved?	High: Highly aware of the data relating to overall quality of services and an understanding of what development is needed. Medium: Aware to less extent. Low: Aware to some extent.
4. Degree of staff involvement	
To what extent are staff involved and prioritised in the production of QI?	High: Staff are fully involved, priorities identified and discussed with staff. Medium: Lower levels of staff involvement, some discussion takes place. Low: Limited involvement.
To what extent are staff involved directly or focused on by the board (eg, in board meeting discussions, agendas)?	High: Staff are involved in, or are the focus of, board discussion and agenda items. Actions and strategies are linked to staff, for example, considering the impact or highlighting the need to canvas opinion. Medium: To some extent. Low: Limited.
5. Degree of public/patient involvement	
To what extent are patients and the public involved and prioritised in QA and QI?	High: Fully involved, priorities identified and discussed with patients/public. Medium: Less involved. Low: Limited involvement.
To what extent are patients involved directly or focused on by the board (eg, in board meeting discussions, agendas)?	High: Patients/public involved in, or are the focus of, board discussions and agenda items. There are actions and strategies in response to the concerns and experiences of patients/public. Medium: To some extent. Low: Limited.
6. Clear, systematic approach	
Are there a manageable number of priorities that are clear and well specified?	High: Small number of priorities, readily apparent, clearly linked to actions. Medium: A larger number of priorities, less clearly articulated in terms of what they are and what the actions are. Low: A large volume of priorities, unclear descriptions and actions.
Are priorities predominantly driven externally?	High: Addressing external requirements while clearly prioritising internally led priorities. Medium: Addressing both external requirements and some internally led priorities. Low: Predominantly driven by external requirements.
7. Balance between clinical effectiveness, patient experience and safety	High: Attending to each aspect equally. Medium: Attending to each aspect but some are more prominent than others. Low: Not all aspects are being dealt with and they are very unbalanced.
8. Dynamics	
How do board members challenge and ask questions of each other?	High: Probing questions alongside supportive comments and advice where relevant. Medium: To a lesser extent. Low: Repetitive questioning and discussion without timely resolution.

QA, quality assurance; QI, quality improvement.

*The aim of quality assurance (QA) is to ensure that minimum standards are being met and to deal with poor performance. It includes mechanisms such as quality monitoring and reporting, national standards, guidelines and targets.

Once the measure was agreed it was used to classify all 15 organisations in our study. Organisations were rated against each item (by LP). Where organisations were considered ‘borderline’ they were given a combined rating (eg, medium/low). An overall rating for each dimension was calculated from the proportional frequencies of the component items (eg, if the majority of items were rated ‘high’ the overall rating for that dimension would be ‘high’). The same process was then used to calculate, for each organisation, an overall classification of QI maturity.

The final stage was a thematic analysis^33^ of the entire data set to illuminate key differences between organisations with different levels of QI maturity. Our analysis thus involved a combination of deductive and inductive forms of inference.^31^ We used the framework that we developed from prior research and existing guidance deductively to guide analysis. We also used an inductive approach to identify additional themes in the data, returning to the literature to further explain our findings.

The study received exemption from NHS Research Ethics processes. Informed consent for interviews was obtained from all participants. Board meetings are held in public, although we informed organisations prior to our attendance of our presence (and our study).

## Results

We found a range of board-level activities related to governance of QI. Boards varied in the extent to which, and the way in which, specific activities were undertaken. Table 3 shows the QI maturity rating for each organisation.

**Table 3:** QI maturity

Organisation	Type	Overall rating	Framework dimensions							
			1	2	3	4	5	6	7	8
1	Benchmarking	High	H	H	H	H	H	H	H	H
2	Participating	High	M	M	H	H/M	H/M	H	H	H
3	Participating	Medium	M	L/M	M	L/M	M	M	M	L
4	Comparator	Low/Medium	M/H	L/M	L/M	L/M	L/M	L/M	L	M
5	Participating	Low	M	L	M	L	L	M	L	L
6	Benchmarking	Medium	M	M	L	M	M	M	M	M
7	Benchmarking	Low	L	L	M	M	H	L	L	L
8	Comparator	Medium	L	M	M	M/H	M/H	L	L	L
9	Participating	Medium	L	M	M	M/H	M/H	L	M	M
10	Participating	Medium/High	M/H	M	H	L/M	L	H	H	M
11	Comparator	High	H	M	H	H	H	M	H	H
12	Comparator	Low/Medium	L/M	L/M	L	M/L	M	L	L	M
13	Comparator	Medium	M	M	M	L	L	M	M	H
14	Comparator	High	H/M	M	H	M/H	H/M	M	H	H
15	Participating	High	M	M	H	M/L	L/M	H	H	H

QI, quality improvement.

### Characteristics of boards with high levels of QI maturity

The following characteristics emerged as particularly important in understanding variation between organisations: QI as a board priority; balancing a short-term (external) focus with a long-term (internal) focus on QI; using data for improvement; patient and staff engagement; and clinical leadership. Additional data relating to each theme are shown in online supplementary table S4.

10.1136/bmjqs-2016-006433.supp1 Supplementary data

### Prioritising QI

Boards with higher levels of QI maturity prioritised QI in their discussions. The priority afforded to QI cannot, however, be inferred simply from the amount of time spent discussing QI as boards with high levels of QI maturity relied on their quality subcommittee to provide detailed scrutiny. These organisations exhibited apparent confidence in the functioning of the subcommittee through, for example, taking the subcommittee report ‘as read’ with only specific items escalated for attention and discussion. This was in contrast to an apparent lack of confidence in the board subcommittee structures within other organisations where the quality committee report was discussed in full at the board meeting. The following extract is from fieldnotes recorded during observation of a board meeting of an organisation rated ‘low’ in relation to QI maturity:

*The report from the quality committee was given by the Nursing Director. The committee has been inquorate for 3 meetings. Not much reported at this point. Appeared there wasn’t a strong handle on the function of this committee and its function was being picked up in the CQC special measures Improvement Plan and other parts of the board meeting and therefore all quality discussion was at the board level.* (Board observation, Organisation 5)

### Balancing a focus on short-term (external) priorities with a long-term (internal) focus on QI

All boards faced multiple external accountability requirements. Boards with high QI maturity addressed these requirements but also had agreed plans to improve quality within the organisation in the long term. In these organisations the Quality Accounts contained clear priorities that were well defined and internally driven. In contrast, in organisations with low QI maturity, Quality Accounts had a large number of priorities that were driven more by external demands, such as national targets for infection control and waiting times for treatment.

One way in which boards with high QI maturity invested in long-term QI was through articulating and enacting values and expected behaviours, and in using these as a basis for staff recruitment. A focus on values was seen by participants to have benefits in terms of the quality of care provided to patients, as well as in encouraging staff to adopt QI processes. Importantly, it was also felt to lead to more effective functioning of the board by enabling trust between board members and managers:

*You can only really rely on information that you get if you trust the people working for you, so that’s why it’s so important for the board. We’re very clear about it in recruitment process, appointment process, revalidation process, and so on. Because trust is an essential ingredient, when clearly the board cannot go through every little detail about whether this patient, or that patient has received the right treatment, the right care, the right behaviours from the staff.* (Interview, chair, Organisation 2)

### Using data for improvement

All boards in this study were confronted with large volumes of data (one board member described this as feeling as if they were ‘drowning in data’). Organisations with high QI maturity received reports where the data were clear, readable, and where different sources of data were discussed together (eg, data on staffing levels considered alongside data on staff well-being and patient experience). Importantly, organisations with a high QI maturity used data for QI, not just assurance. For example, data were linked to actions and these actions were monitored. In contrast, reports to boards with low QI maturity were characterised by a large volume of data, often not clearly presented, reviewed in silos and not linked to improvement actions.

Boards with high QI maturity used a range of types of data. In one example from our study the chair used unannounced visits to wards to detect potential problems. In this organisation the Chief Executive also personally read all complaint letters. This type of ‘soft intelligence’^34^ was seen to be important for detecting problems and identifying areas for improvement. It was also considered to be a valuable source of insight into the nature of problems that could then be used as a guide for action.

Reports to the boards with high QI maturity used ‘benchmarks’ and carefully identified relevant comparators. In contrast, organisations with a low QI maturity were less likely to compare themselves with others, and where benchmarking data were used these were considered in isolation (from other metrics) and used mainly for external reporting requirements.

### Patient and staff engagement

Boards with a high QI maturity made systematic efforts to collect the views and experiences of staff and patients and involve staff and patients in the development of strategy. In these organisations staff well-being was a noticeable inclusion in the board agenda and the experience and views of staff and patients were a ‘common thread’ through board agenda items. For example, in one organisation with high QI maturity, an away day was held with clinical staff to develop the organisation’s strategy. In this organisation board members also attended meetings with patient groups and, importantly, translated the issues that were raised in discussion into actions:

*We have something called a patient experience stakeholder forum which is a combination of staff, patients, governors, Health Watch, people who would have a whole view and then what we’ve been doing is feeding back to them. So they agreed our strategy, they have revised our plan of action because they didn’t agree with the focus and timing.* (Interview, Director of Nursing, Organisation 2)

### A culture of continuous improvement

Organisations with high QI maturity were characterised by constant questioning and self-examination. In these organisations ‘striving for excellence’ was seen, and promoted, as part of the corporate identity. For example, in one board meeting, following a discussion of current performance on staff engagement, the chair stated that ‘*we don’t do average. We want to be the best out there*’ (Board Observation, Organisation 1). A key feature of organisations with high QI maturity was a culture that supported learning and improvement. These organisations used external networks for learning, proactively discussing particular issues with staff from regulatory agencies, researching how other hospitals had responded to similar problems and visiting high-performing organisations. For example, during a discussion at a board meeting on a particular performance metric (theatre utilisation) the chair asked ‘*who is good at it, who could we talk to?*’ Board members from this organisation also visited high-performing organisations to see what they could learn and reported back to the board with initiatives that could potentially be implemented locally (Board Observation, Organisation 1).

In contrast, organisations with low QI maturity appeared more complacent than those with higher levels of QI maturity. At times there was also insufficient challenge from non-executive directors. In these organisations executive directors were observed to bring overly optimistic trajectories of future performance to the board or to put a ‘positive spin’ on poor performance. During one board observation this practice was picked up by a newly appointed chair, as can be seen in the following reprimand from the chair to an executive:

*…the nursing trajectory is optimistic. I think what comes back to the board is our best realistic thinking not an element of wishful thinking.* (Board Observation, Organisation 7)

### Clinical leadership

It was notable that the boards of organisations with high QI maturity had a higher number of clinicians on the board (three or more) than other organisations. Moreover, in these organisations the medical director and the director of nursing had an extended role that included an additional corporate function, such as ‘director of governance.’ In organisations with high QI maturity, such clinical leaders were observed to be visible and vocal, communicated assertively and were apparently well regarded by other board members (as suggested through verbal and non-verbal reactions). They were also observed to provide the board with helpful analysis of quality and safety concerns, for example, by explaining trends, both positive and negative, and why issues had arisen. They made connections between different sources of data (eg, between data on staffing levels, staff well-being and patient experience) and supplemented quantitative data with ‘softer’ intelligence gleaned from time spent on the ward.

In contrast, the clinical leaders on the boards of organisations with low QI maturity made very little overall contribution to the meeting, speaking only to a small number of items related to their clinical remit. We also recorded instances of there being no medical director in post (and no interim provision) (Organisation 6), of unhelpful responses from the medical director to questions from the chair (Organisation 10), and clinical leaders who spoke so quietly that the chair, or members of the public, had to request that they speak louder (Organisation 5, Organisation 7). We also observed an instance where clinical leadership was enacted in what we considered to be a largely rhetorical, rather than a substantive, way. For example, one director of nursing spoke at length about her familiarity with front-line patient care, and her concern for quality, but in such a way as to discourage further challenge from non-executive members, potentially inhibiting the effectiveness of the board (Organisation 10).

There is an indication—from interviews and board observations—that clinical leaders in organisations with high levels of quality governance maturity had also built relationships with external partners, such as commissioners and regional NHS managers, and brought to the board knowledge of the external policy environment. However, there was also the suggestion that medical directors, in particular, attended numerous external planning meetings, potentially impacting on their role within the organisation, which requires further research. This is alluded to in the following admonishment from a chair to the board:

*The top corridor is empty quite a lot of the time. I take it because you are all in these meetings in [name of locality], which I find annoying.* (Chair, Organisation 9)

## Discussion

We used a framework developed from previous research and existing guidance to analyse the activities of hospital boards in England to develop a measure of a maturity in relation to governance of QI (QI maturity). We applied this measure to the organisations in our study and then explored the characteristics of boards with differing levels of QI maturity. We found that organisations with higher levels of QI maturity prioritised QI, balanced attention to short-term (external) priorities with a long-term (internal) investment in QI, used data for QI, not just QA, engaged staff and patients in QI and had a culture of continuous improvement. These characteristics often appeared to be enabled and facilitated by clinical leaders. We contribute to the literature on board governance of quality by describing how these characteristics were enacted.

Our study highlights the apparent importance of board-level clinical-manager ‘hybrids’ in QI.^35^ Previous research has found an association between high levels of clinician involvement in hospital boards and higher quality of care.^36 37^ Findings from a longitudinal analysis by Veronesi *et al* 37 suggest that it is clinical involvement in boards that is contributing to performance and not the reverse (high-performing organisations recruiting more clinicians at senior levels). However, little is known about *how* clinicians on the board improve quality.

We found that in organisations with a high QI maturity clinical leaders brought in-depth knowledge and understanding of quality issues and provided the board with meaningful analyses of data. Recent research has emphasised the importance of meaningful representation and interpretation of data by boards.^38^ Medical directors, in particular, appeared to contribute important translation work, using knowledge and skills drawn from their medical training, or from dedicated training in QI. Clinical leaders also contributed knowledge of relevant developments in national policy and links to external networks. Previous research has highlighted the importance of good relationships with external partners, to access external resources; facilitate learning; and to negotiate, shape and align external regulatory and performance management demands with internal priorities for QI.^39 40^

In organisations with high QI maturity clinical leaders thus appear to play a critical role as ‘boundary spanners,’^41^ providing a link between ‘the board and the ward,’ making connections between data silos and aligning external demands with internal priorities. Although all organisations in our study had board members with clinical backgrounds, in those with high QI maturity clinical leadership was a state of *doing* rather than *being*. In these organisations clinical leaders were observed to be active in making connections and contributing to the decision making of the unitary board. While clinicians have always held leadership positions in the NHS, previous research suggested that they tended to adopt an advocacy position, promoting the interests of the profession or their own specialty.^14^ In contrast, our research suggests that, in organisations with high QI maturity, clinical leaders are orientated to strategic decision making, and to a corporate goal of QI. Further research is needed to confirm and elaborate these findings.

This research had some limitations. We only observed the public part of the board meeting. We did not observe board subcommittees. The use of an overall three-point classification system (L/M/H) could mask potential variation within organisations between the different dimensions of board governance of QI. It also only relates to board practices, rather than the organisation as a whole. Our development of a measure of quality governance maturity was exploratory and our assumptions may be open to debate; however, we undertook the task systematically, used the entire data set, and our methods are explicit and transparent. We grounded our analysis in both the data and the literature.

## Conclusions

Boards of healthcare organisations with high QI maturity prioritised QI; combined a focus on short-term (external) priorities with long-term (internal) investment in QI; used data for QI, not just assurance; engaged staff and patients; and had a culture of continuous improvement. These characteristics appeared to be facilitated by clinical leaders. Future research should explore the biographies, identities and work practices of board-level clinical leaders and their role in organisation-wide QI.

We have developed a measure of QI maturity to help us understand how boards of healthcare organisations enact governance of QI, and to compare organisations within our study. There is potential for the measure to be further developed and applied formatively to assess, or self-assess, and improve an organisation’s governance of QI.
