# Supplementary material for: How do hospital boards govern for quality improvement? A mixed methods study of 15 organisations in England
Source: BMJ Qual Saf. 2017 Jul 8;26(12):978–86. doi: 10.1136/bmjqs-2016-006433 (PMC5750431; doi:10.1136/bmjqs-2016-006433)
Supplement: Supplementary data [file bmjqs-2016-006433supp001.docx]

**Table 4. Differences between organisations with a high and low QI maturity. Illustrative data for each theme**.

|  | High | Low |
| --- | --- | --- |
| Prioritising QI | The first item on the agenda is ‘QI strategy’. (Documentary analysis, board minutes, Organisation 11).  Quality part of the agenda was 65 mins (of total board meeting duration of 2 hours), QI related elements totalled c.26 mins (remainder focused on performance-related discussion). (Board observation, Organisation 1)  Appeared to be fairly good devolvement to committee structures. Report was given to the board but was taken as read, with highlights and Q&A. (Board observation, Organisation 1).  Chair noted that ‘it was impossible for the board to be involved in an individual case. Need to make sure leadership and management team are dealing with it, and where the system is not working well, training is given. That is as far as the board can go. We will leave you [committee/exec] alone to deal with it and we want to know what progress is made over time’*.* (Board observation, Organisation 1) | All matters appeared to be dealt with at the board meeting. No confidence or structure in place for sub-committees to deal with items. (Board observation, Organisation 7)  Quality and safety early in the agenda but after all the external reports – Monitor, CQC, Keogh declaration, KPMG action plan etc. (Documentary analysis, board minutes, Organisation 7)  The report from the quality committee was given by the Nursing Director. The committee has been inquorate for 3 meetings. Not much reported at this point. Appeared there wasn’t a strong handle on the function of this committee and its function was being picked up in the CQC special measures Improvement Plan and other parts of the board meeting and therefore all quality discussion was at the board level. (Board observation, Organisation 5). |
| Balancing a short-term (external) focus with a long term (internal) focus on QI | ‘If you get people whose mindset is caring, and being candid, they’re more likely to adopt the quality processes and procedures that you require.’ (Interview, chair, Organisation 2)  ‘You can only really rely on information that you get if you trust the people working for you, so that’s why it’s so important for the board. We’re very clear about it in recruitment process, appointment process, revalidation process, and so on. Because trust is an essential ingredient, when clearly the board cannot go through every little detail about whether this patient, or that patient has received the right treatment, the right care, the right behaviours from the staff.’ (Interview, chair, Organisation 2)  Prioritising more than ‘must do’s’. Strong sense of owning and executing a QI agenda for their organisation with national targets being a part of it but not the main driver. (Documentary analysis, Quality Account, Organisation 1) | ‘To be honest we’re sitting in an organisation where there has been, to save cash in the past, a disinvestment in the clinical governance team. So 9 or 10 posts were taken out that have never been put back in. We don’t have an electronic patient record, we don’t have order comms for the labs, we’ve only just gone onto an electronic risk system really, and started to use that. So we’re not sitting in an organisation that’s saying well actually we’ve got a pretty good clinical governance structure, we’ve got the right sort of people in place with the time to develop these things. We’ve got a team that pretty much is fighting to keep its head above water just doing, you know.’ (Interview, Medical Director, Organisation 5)  Trust in special measures, just focused on short term priorities. (Board observation, Organisation 7) |
| Using data for QI | ‘I read every complaint which is actually fascinating in looking at how your services work. So I think that’s quite important to do that and it also gives you some immediate, it does give you very good insight into when things go wrong, and that maybe because it went wrong on that day, or there may be a sort of inherent problem within an area. So it’s not unusual for me to pick things up from those complaint letters even pre the investigation. Because the complaint letter you can often read them and you either know there’s a story here, there’s a problem, or not, it’s fairly clear, and well yes, of course, we do have some more detailed analysis, but also if you read them you begin to pick up the cluster.’ (Interview, CEO, Organisation 2)  ‘I spend quite a lot of time in the Trust, in the hospital in community centres and so on, just sniffing around.’ (Interview, chair, Organisation 2)  ‘And clearly if you’ve got wards which don’t have enough trained people on them to do the job properly it’s very hard to improve quality.’ (Interview, NED, Organisation 2)  A lot of benchmarking, particularly in data presentation. Data presented alongside other organisations’ data. (Board observation, Organisation 2)  QI data is linked to other data, e.g. the QI dashboard shows sickness rates and in the commentary this is linked to patient experience. (Board observation, Organisation 1) | Data presented in silos– not linked. (Board observation, Organisation 7)  Very difficult to read the data in meeting papers. Small font. Huge amount of information in the tables. (Board observation, Organisation 7).  No mention of benchmarking. (Documentary analysis, board minutes, Organisation 12)  Not much mention of benchmarking in the board discussions. (Board observation, Organisation 4) |
| Patient and staff engagement | ‘We have something called a patient experience stakeholder forum which is a combination of staff, patients, governors, Health Watch, people who would have a whole view and then what we've been doing is feeding back to them. So they agreed to our strategy, they have revised our plan of action because they didn’t agree with the focus and timing.’ (Interview, Director of Nursing, Organisation 2)  All items discussed are brought back to the relevance to patients and staff and/or what do patients and staff think. (Board observation, Organisation 11)  There is a clear QI programme - specifically states ‘led by frontline staff, patients and governors’. (Documentary analysis, Quality Account, Organisation 11) | ‘I mean for instance if you’re looking, you know, at the board level, the use of patients coming in to tell their stories is something that we just haven’t really embraced at all.’ (Interview, Medical Director, Organisation 5)  A lot of commentary about the importance of patient experience but no clear involvement of patients in establishing the priorities. (Documentary analysis, Quality Account, Organisation 8) |
| Continuous improvement culture | ‘We have in some of our performance data, we know where that puts us against other hospitals of our kind, when we get our staff survey, when we get our patient surveys, we pick a group so we know where we are standing against, other Trusts in England, other Trusts in London, so we do that sort of benchmarking. In the last twelve months the Director of Nursing and I, we’ve been on two visits where we think there’s been good work done, so one’s to [name of organisation] about the work they’re doing on their wards that really engage staff, where staff become excellent around their training and development. And their care and their engagement scores are phenomenal.  INT: Are they?  Yeah, really positive, it was really excellent to see it….and the other one was to [name of organisation] to look at what they’ve done around remodelling their outpatients appointments, outpatients department so you get a better service and to improve the quality, and that was really impressive, but in both cases we thought we can come back and learn from them and try and develop our approach.’ (Interview, Medical Director, Organisation 1)  The Executive Nurse Director had an informal meeting with CQC to discuss never events. (Board observation, Organisation 11)  During a discussion on a performance metric (theatre utilization) the chair asked ‘who is good at it, who could we talk to?’ Board members also visited a high performing Trust to see what they could learn and reported back to the board with initiatives that could potentially be implemented locally. (Board observation, Organisation 1)  Following a discussion of current performance on staff engagement, which included benchmarking with other organisations, the chair stated that ‘we don’t do average. We want to be the best out there’. (Board observation, Organisation 1) | ‘The Buddy Trust has only just really been announced. And we’re only just getting round to asking them, you know, what they could do to help. So seven months in that’s where we’re at.’ (Interview, Interim Medical Director, Organisation 7)  The Director of Human Resources reported on an initiative to recruit nurses from the Philippines. The original goal was 60, this was then revised to 40. However only 5 have so far been through language training. The Director of Human Resources referred to an anecdote from a single nurse, from Spain, who said that ‘people in Europe see the language test as daunting and very academic’. The executive appears to me to lack strategy and to be inappropriately positive about the situation. There is no evidence of benchmarking or learning from what other organisations are doing. My impressions were echoed by comments from the (newly appointed) chair who stated that: ‘…the nursing trajectory is optimistic. I think what comes back to the board is our best realistic thinking not an element of wishful thinking’. (Board observation, Organisation 7) |
| Clinical leadership | ‘My role is Chief Nurse and Director of Governance. I'm responsible for both clinical and corporate governance currently in the trust but of course I'm a nurse by background, so Chief Nurse is my main raison d'etre for the board.’ (Interview, Chief Nurse, Organisation 2)  ‘…then (the Medical Director) has just arrived and of course he’s very strong on improvement technique and quality improvement and so on.’ (Interview, Chief Nurse, Organisation 2)  ‘I think the other bit of context is that over the last six years maybe a bit more, we’ve really invested in clinical leadership, we've really moved to a very clinically led organisation, so there are lots of clinicians, mainly doctors, but not just doctors, appointed in management positions.’ (Interview, Medical Director, Organisation 15) | Report from the Quality and Safety Committee is given by the Chief Nurse (no other corporate role). The chair asked the Chief Nurse to speak up. The Chief Nurse reported data on the incidence of falls, pressure ulcers and serious incidents, but not in a way that was meaningful. For example, the Chief Nurse reported 5 serious incidents stating only that there would be ‘root cause analysis and any learning followed through’. The emphasis was on QA not QI. (Board observation, Organisation 7)  Item 6. Strategy and Planning: In a discussion about progress with partnership working on the Accountable Care Organisation the medical director referred to this is as a ‘significant commitment of time’. (Board Observation, Organisation 5) |
